# Effect of Exposure to Atmospheric Ultrafine Particles on Production of Free Fatty Acids and Lipid Metabolites in the Mouse Small Intestine

**DOI:** 10.1289/ehp.1307036

**Published:** 2014-08-29

**Authors:** Rongsong Li, Kaveh Navab, Greg Hough, Nancy Daher, Min Zhang, David Mittelstein, Katherine Lee, Payam Pakbin, Arian Saffari, May Bhetraratana, Dawoud Sulaiman, Tyler Beebe, Lan Wu, Nelson Jen, Eytan Wine, Chi-Hong Tseng, Jesus A. Araujo, Alan Fogelman, Constantinos Sioutas, Mohamed Navab, Tzung K. Hsiai

**Affiliations:** 1 Division of Cardiology, Department of Medicine, School of Medicine, University of California, Los Angeles, Los Angeles, California, USA; 2 Department of Biomedical Engineering, and; 3 Department of Civil and Environmental Engineering, University of Southern California, Los Angeles, California; 4 Department of Bioengineering, School of Engineering and Applied Science, University of California, Los Angeles, Los Angeles, California, USA; 5 Division of Pediatric Gastroenterology, Departments of Pediatrics and Physiology, University of Alberta, Edmonton, Alberta, Canada

## Abstract

Background: Exposure to ambient ultrafine particulate matter (UFP) is a well-recognized risk factor for cardiovascular and respiratory diseases. However, little is known about the effects of air pollution on gastrointestinal disorders.

Objective: We sought to assess whether exposure to ambient UFP (diameter < 180 nm) increased free fatty acids and lipid metabolites in the mouse small intestine.

Methods: *Ldlr*-null mice were exposed to filtered air (FA) or UFP collected at an urban Los Angeles, California, site that was heavily affected by vehicular emissions; the exposure was carried out for 10 weeks in the presence or absence of D-4F, an apolipoprotein A-I mimetic peptide with antioxidant and anti-inflammation properties on a high-fat or normal chow diet.

Results: Compared with FA, exposure to UFP significantly increased intestinal hydroxyeicosatetraenoic acids (HETEs), including 15-HETE, 12-HETE, 5-HETE, as well as hydroxyoctadecadienoic acids (HODEs), including 13-HODE and 9-HODE. Arachidonic acid (AA) and prostaglandin D_2_ (PGD_2_) as well as some of the lysophosphatidic acids (LPA) in the small intestine were also increased in response to UFP exposure. Administration of D-4F significantly reduced UFP-mediated increase in HETEs, HODEs, AA, PGD_2_, and LPA. Although exposure to UFP further led to shortened villus length accompanied by prominent macrophage and neutrophil infiltration into the intestinal villi, administration of D-4F mitigated macrophage infiltration.

Conclusions: Exposure to UFP promotes lipid metabolism, villus shortening, and inflammatory responses in mouse small intestine, whereas administration of D-4F attenuated these effects. Our findings provide a basis to further assess the mechanisms underlying UFP-mediated lipid metabolism in the digestive system with clinical relevance to gut homeostasis and diseases.

Citation: Li R, Navab K, Hough G, Daher N, Zhang M, Mittelstein D, Lee K, Pakbin P, Saffari A, Bhetraratana M, Sulaiman D, Beebe T, Wu L, Jen N, Wine E, Tseng CH, Araujo JA, Fogelman A, Sioutas C, Navab M, Hsiai TK. 2015. Effect of exposure to atmospheric ultrafine particles on production of free fatty acids and lipid metabolites in the mouse small intestine. Environ Health Perspect 123:34–41; http://dx.doi.org/10.1289/ehp.1307036

## Introduction

Exposure to ambient fine particulate matter (PM_2.5_; diameter ≤ 2.5 μm) is associated with cardiovascular and pulmonary diseases, cancer, and stroke (Brook et al. 2010; Brunekreef and Holgate 2002; Gorham et al. 1989; Villeneuve et al. 2006). Ultrafine particles (UFP; diameter ≤ 180 nm) are a subfraction of PM that mostly originate from vehicular emissions, and are highly enriched in redox-active cycling organic chemicals (Sardar et al. 2005). UFP also harbor a higher level of oxidizing potential than do larger particles (Nel et al. 2006; Zhang et al. 2008) to reduce the anti-inflammatory capacity of high-density lipoprotein (HDL) and to accelerate atherosclerosis (Araujo et al. 2008; Brook et al. 2010; Pope et al. 2004). Whether UFP are implicated in gastrointestinal disorders is of increasing clinical interest.

A number of emerging studies support air pollution as an environmental risk factor for inflammatory bowel diseases (IBD) and appendicitis (Kaplan 2011; Kaplan et al. 2009, 2010, 2013). Whereas acute air pollution exposure was reported to induce nonspecific abdominal pain in one study (Kaplan et al. 2012), chronic exposure to high levels of manganese in air pollution was also associated with increased mortality in hepatic disease (Spangler 2012). It has been postulated that gastrointestinal exposure to air pollutants occurs via mucociliary clearance of PM from the lungs as well as ingestion via food and water sources (Beamish et al. 2011). Plausible mechanisms include the direct effects of particulate pollutants on epithelial cells, resulting in systemic inflammation and immune activation as well as modulation of the intestinal microbiota (Kreyling et al. 1999; Möller et al. 2004). In this context, assessing the metabolic mechanisms whereby ambient PM regulates the digestive system is of potential clinical significance.

Recently, exposures to ambient PM and diesel exhaust were associated with a reduction in the antioxidant and anti-inflammatory capacities of HDL in *ApoE*-null mice (Araujo et al. 2008; Yin et al. 2013). UFP further modulated lipid metabolism and the antioxidant property of HDL, while administration of D-4F, an apolipoprotein A-I (ApoA-I) mimetic peptide, attenuated UFP-modulated lipid metabolism and atherosclerosis (Li et al. 2013), which is consistent with studies demonstrating that D-4F restored HDL function and attenuated atherosclerosis (Morgantini et al. 2011; Navab et al. 2002). In this context, we postulated that exposure to ambient UFP modulates intestinal lipid metabolisms. Our studies revealed that mice exposed to UFP, collected near downtown Los Angeles, California, displayed increased oxidative products of arachidonic and linoleic acids, accompanied by changes in villus lengths and macrophage/neutrophil infiltrates in the intestinal villi, whereas D-4F administration mitigated these effects. Thus, these findings provide an animal model to further elucidate the mechanisms whereby air pollution modulates lipid metabolism and inflammatory responses in the digestive system.

## Materials and Methods

*Ultrafine particle collection*. Size-fractionated urban particulate pollutants were collected at the University of Southern California (USC) campus near downtown Los Angeles, California. The UFP represent a mixture of pollution sources, including fresh ambient PM from areas impacted by heavy-duty diesel trucks, light-duty gasoline vehicles and ship emissions, as well as PM generated by photochemical oxidation of primary organic vapors (Verma et al. 2009b). Briefly, UFP were collected by a High-Volume Particle Sampler on Zefluor PTFE Membrane filters (3 μm, 28139-597; Pall Life Sciences), as previously described (Misra et al. 2002). The collected PM samples were extracted from the filter substrates by soaking in ultrapure Milli-Q water for 30 min followed by 5 min of vortexing and 30 min of sonication. The aqueous suspension was reaerosolized for *in vivo* exposure experiments with a Vortran nebulizer (Vortran Medical Technology Inc.) and diffusion dried (Diffusion Dryer 3062; TSI Inc.) with the static charges removed using a neutralizer before entering the exposure chambers, using methods previously described in detail by Morgan et al. (2011).

*Mouse exposure to UFP*. Humane care and use of animals were observed to minimize distress and discomfort. A maximum of five mice were housed per shoebox-type cage with solid flooring and woodchip bedding in the USC vivarium (Ray R. Irani Building). The housing conditions were maintained at the seminatural light cycle of 12:12 hr light:dark and at a temperature of 23°C and humidity of 55 ± 15%. Mice had constant access to food and water except for the duration of exposure.

Exposure 1. Mice were exposed to reaerosolized UFP or HEPA-filtered air (FA; used as control) via whole-body animal exposure chambers. The size distribution and number concentration of the highly concentrated reaerosolized UFP were monitored using TSI 3080 Scanning Mobility Particle Sizer and TSI 3022 Condensation Particle Counter. Particle mass concentration and UFP composition were measured by collecting particles on 37-mm Teflon filters (PTFE 2 μm; Gelman Science) and 37-mm precleaned quartz filter (Pallflex Corp). Teflon filters were assayed for quantification of UFP water-soluble trace elements and metals, using a magnetic sector inductively coupled plasma mass spectrometry (ICP-MS) method, as described in Herner et al. (2006). Organic and elemental carbon were also quantified by the NIOSH (National Institute for Occupational Safety and Health) thermal optical transmission method performed on the quartz filters, as described by Schauer (2003) and Sullivan et al. (2004).

Animal exposure studies were performed in compliance with the University of Southern California IACUC (Institutional Animal Care and Use Committee) protocol. Mice were acclimated for 2 weeks before the start of the exposure studies. Age- and weight-matched *Ldlr*-null mice (at the age of 90 days and an average weight of 24.8 ± 1.5 g) under the C57BL/6 background (stock #002207; Jackson Laboratory) were grouped randomly, and exposed to ambient FA or UFP for 5 hr/day (1030–1530 hours), 3 days/week for 10 weeks in the whole-body exposure chambers and were placed on a high-fat diet (HFD-D12492: 5.24 kcal/g, 34.9 g% fat, 26.2 g% protein, 26.2 g% carbohydrate; Research Diets). In parallel, a Scanning Mobility Particle Sizer (SMPS Model 3080; TSI Inc.) was used to monitor particle size distribution and exposure concentrations. The resuspended aerosol size distribution approximated airborne PM measured at the USC site as previously described (Verma et al. 2009a). The time-averaged exposure mass and number concentrations were approximately 360 (± 25) μg/m^3^ and 2.4 (± 0.17) × 10^5^ particles/cm^3^.

D-4F (synthesized and purified by Peptisyntha Inc., Torrance, CA) was initially administered via subcutaneous injection at 0.2 mg/mouse/day dissolved in phosphate buffered saline (PBS); PBS alone was injected as the control. After the first week, D-4F was administered daily via drinking water at 0.2 mg/mL, and each mouse had about 4 mL of water intake on average (about 800 μg D-4F/day/mouse). On the exposure day, D-4F was administered after the mice were returned to housing cages. Five mice were initiated in each group. One mouse died in each group as a result of subcutaneous injection, fighting, or wound infection. These groups of mice were previously assessed for serum HDL antioxidant capacity and atherosclerotic lesion size (Li et al. 2013).

Exposure 2. To exclude the possibility that the increased lipid peroxidation and inflammatory responses from the first exposure study was influenced by the high-fat diet, we performed a second UFP exposure study to corroborate the intestinal lipid oxidation by feeding the animals on a normal chow diet. *Ldlr*-null mice (*n* = 6/group) were exposed to FA, FA+D-4F, UFP, and UFP+D-4F; FA with D-4F administration served as an additional control group.The mice were fed on the normal chow diet (Testdiet), and D-4F was administered via the drinking water. The time-averaged exposure mass and number concentrations were 466 (± 39.4) μg/m^3^ and 4.5 (± 0.31) × 10^5^ particles/cm^3^, respectively.

**Figure 1:**
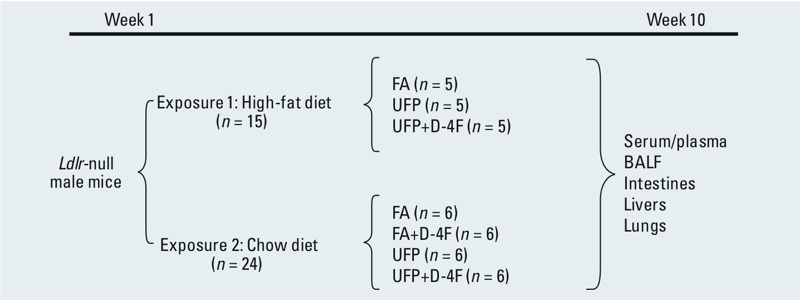
Illustration of exposure experiments. BALF, bronchoalveolar lavage fluid.

The exposure experiments are illustrated in Figure 1.

*Quantification of lipid metabolites*. Mice were euthanized with inhalation of isofluorane on 2 consecutive days (FA groups on day 1 and UFP groups on day 2) immediately after completion of the 10-week exposure. Blood (0.2–0.4 mL) was drawn from the left eye for plasma preparation using plasma separators (BD Biosciences) as previously described (Navab et al. 2012). Measurements of lipid metabolites in plasma were performed as previously described (Li et al. 2013; Navab et al. 2012). The small intestines were dissected and rinsed with cold saline, and the outer intestinal fat was removed. The intestines and liver tissues were cut into small pieces (1–2 mm) in PBS with 20 μM butylhydroxytoluene (BHT) and were then homogenized. After centrifugation at 13,200 rpm for 20 min at 4°C, about 75 μL of the supernatant was collected for mass spectrometry (4000 QTRAP; Applied Biosystems) (Buga et al. 2010; Imaizumi et al. 2010). The levels of oxidative products of arachidonic and linoleic acids [hydroxyeicosatetraenoic acids (HETEs) and hydroxyoctadecadienoic acids (HODEs)], as well as arachidonic acids (AA), prostaglandin D_2_ (PGD_2_), and lysophosphatidic acid (LPA) were determined by liquid chromatography, electron spray ionization, and tandem mass spectrometry (LC-ESI-MS/MS) as previously described (Buga et al. 2010; Imaizumi et al. 2010). In each instance, a deuterium-labeled internal standard was included to correct for extraction efficiency and to facilitate quantification.

*Measurement of SAA and TNF-*α *levels*. Plasma levels of serum amyloid A (SAA) were determined using the ELISA kit from Invitrogen as previously described (Navab et al. 2012), and plasma levels of tumor necrosis factor-α (TNF-α) were determined using the ELISA kit from BioLegend using the same plasma for lipid analysis.

*Histology and immunohistochemistry*. Ileum segments were collected at the same time as plasma and other tissues, and were fixed in PBS/4% paraformaldehyde and embedded in paraffin blocks. Hematoxylin and eosin (H&E) staining of 5 μm cross-sections was performed to assess changes in the villus morphology. Macrophages and neutrophils were stained with F4/80 antibody (Invitrogen; diluted at 1:100) and Ly6G antibody (Biolegend; diluted at 1:100), respectively, as described by Burns et al. (2012).

Villus lengths were calculated from the pixel lengths of multiple well-oriented representative villi for each small intestinal cross-section using a 10-μm scale. Pixel lengths of small intestine villi were measured on the H&E-stained cross-sections by using ImageJ software (http://imagej.nih.gov/ij/). Quantification was performed by drawing a line that covers the length of each villus and then by using the “Analyze > Set Scale” function of the program to determine the length in pixels. The numbers of macrophages per cross-section were counted and averaged to macrophages per villus. In the second exposure experiment, we quantified macrophage and neutrophil staining by the intensity of F4/80 antibody and Ly6G antibody with ImageJ software. Briefly, each image was thresholded to determine the area of staining, then substracted from the original image to remove the background. Using “Analyze > Set Measurements” in the ImageJ program allowed for quantification of the grayscale intensities of the resulting image to yield the intensity of the antibody staining

*Bronchoalveolar lavage fluid (BALF) collection and analyses*. To examine the pulmonary response of UFP exposure, we collected and analyzed BALF. For detailed methods, see Supplemental Material, “Materials and Methods” sections “BALF collections and cell analyses” and “BALF chemical and cytokine analyses.”

*Statistical analyses*. Data were expressed as mean ± SD unless otherwise stated. Multiple comparisons were performed by one-way analysis of variance, and statistical significance for pairwise comparison was determined by Tukey post test. A *p*-value < 0.05 was considered statistically significant. We further performed effect sizes in our power analyses to determine a number of lipid metabolites in response to UFP exposure and D-4F administration.

## Results

*Characteristics of UFP*. The UFP collected from the USC campus near downtown Los Angeles were reaerosolized using a nebulizer before the mice were exposed, as previously reported (Morgan et al. 2011). The size distribution is similar to those in previously reported studies in the same location (Verma et al. 2009a), with number-based average size < 100 nm for both exposures (see Supplemental Material, Figure S1). The main chemical constituents in UFP were analyzed in terms of their organic and elemental carbon content as well as water-soluble trace element and metal species (Table 1). Although the specific chemical components of PM implicated in adverse health outcomes remain to be defined, UFP harbored elevated proportions of oxidatively active compounds such as organic and elemental carbon, and redox active trace elements and metals including iron, copper, nickel, manganese, and vanadium, as previously reported (Verma et al. 2009a).

**Table 1:** Chemical composition of UFP (mean ± SD).

Exposure	Exposure 1	Exposure 2
Carbonaceous components (μg/mg)
Organic carbon	201.91 ± 16.8	192.3 ± 30.3
Elemental carbon	11.1 ± 2.02	4.62 ± .68
Water-soluble trace element and metal species in UFP (ng/mg)
Lithium	35.2 ± 19	12.86 ± 0.66
Boron	82.3 ± 33.9	74.86 ± 8.32
Sodium	35373.1 ± 1436.9	28032.46 ± 489.9
Magnesium	4958.8 ± 76.2	6032.23 ± 181.89
Aluminum	488.7 ± 154.8	478.71 ± 22.46
Phosphorus	441.4 ± 182.3	280.71 ± 67.13
Sulfur	52910.6 ± 3094.4	46928.51 ± 986.73
Potassium	9366.7 ± 1730.7	4021.28 ± 103.59
Calcium	28591.6 ± 3756.9	26355.27 ± 1729.06
Scandium	0.035 ± 0.016	0.18 ± 0.71
Titanium	1.8 ± 0.5	2.4 ± 2.37
Vanadium	106.1 ± 23.6	13.06 ± 0.84
Chromium	16.8 ± 4.4	19.99 ± 1.78
Manganese	287.1 ± 25.4	129 ± 4.12
Iron	283.2 ± 34.8	228.57 ± 17.46
Cobalt	2504.4 ± 2326.6	35.06 ± 1.45
Nickel	82.3 ± 6.8	142.45 ± 9.97
Copper	1111.7 ± 220.4	439.93 ± 12.91
Zinc	2675.6 ± 390.8	2160.19 ± 40.25
Arsenic	22.6 ± 8.2	9.82 ± 0.78
Rubidium	10.4 ± 1.7	4.31 ± 3.22
Strontium	274.4 ± 62.7	237.74 ± 2.79
Yttrium	0.14 ± 0.02	0 ± 0.07
Niobium	0.047 ± 0.017	0.02 ± 0.09
Molybdenum	44.5 ± 13.1	32.56 ± 1.2
Rhodium	0.19 ± 0.029	0.04 ± 0.02
Palladium	2.4 ± 0.4	0.56 ± 0.3
Silver	0.56 ± 0.06	NA
Cadmium	8.5 ± 0.7	2.83 ± 0.74
Tin	19.2 ± 9.2	6.39 ± 3.11
Antimony	126.6 ± 31.8	45.05 ± 0.89
Cesium	0.89 ± 0.056	0.36 ± 0.07
Barium	800.3 ± 223.3	341.72 ± 9.76
Lanthanum	0.3 ± 0.0439	0.23 ± 0.07
Cerium	0.27 ± 0.03	0.36 ± 0.08
Praseodymium	0.03 ± 0	0.03 ± 0.05
Neodymium	0.2 ± 0.073	0.18 ± 0.08
Samarium	0.14 ± 0.05	0.04 ± 0.02
Europium	3.74 ± 0.92	0.18 ± 0.02
Dysprosium	0.023 ± 0.007	0.03 ± 0.03
Holmium	0.006 ± 0.002	0.01 ± 0.01
Ytterbium	0.016 ± 0.001	0.02 ± 0.02
Lutetium	0.003 ± 0.001	0.0024 ± 0.0035
Tungsten	3.902 ± 1.058	3.93 ± 0.3
Platium	0.055 ± 0.017	0.01 ± 0.05
Thallium	0.81 ± 0.057	0.24 ± 0.04
Lead	25.9 ± 4.01	18.59 ± 0.62
Thorium	0.04 ± 0.003	0.18 ± 0.19
Uranium	0.072 ± 0.017	0.12 ± 0.03
NA, not applicable.		

**Figure 2:**
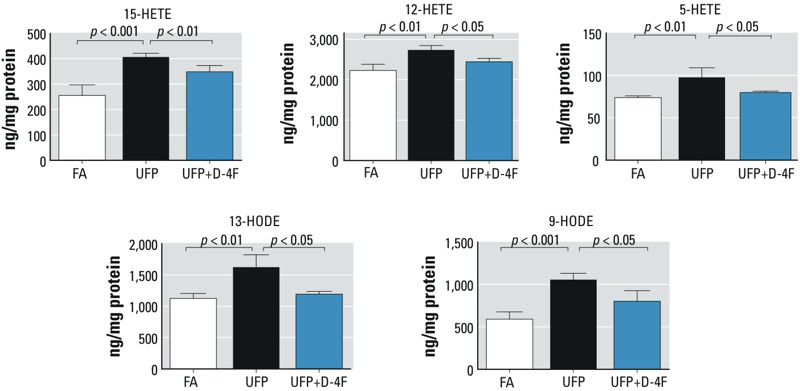
UFP exposure increased intestine levels of HETEs and HODEs, which were attenuated by D-4F administration. *Ldlr*-null mice were exposed to control FA or UFP for 5 hr/day, 3 days/week, for 10 weeks. Lipids were extracted from the intestine, and the levels of HETEs and HODEs were measured by LC-ESI-MS-MS (exposure 1). UFP exposure significantly increased intestinal levels of 15-HETE, 12-HETE, 5-HETE, 13-HODE, and 9-HODE (*p* < 0.001 to *p* < 0.01, *n* = 4). Administration of D-4F significantly reduced these increases (*p* < 0.01 to *p* < 0.05, *n* = 4).

*UFP exposure increased intestinal HETEs and HODEs*. HETEs and HODEs are the oxidative metabolites of arachidonic and linoleic acids, respectively, which are intimately involved in atherogenesis (Funk and Cyrus 2001; Imaizumi et al. 2010). HETEs also appear to modulate colonic inflammation and colorectal cancer risk (Shureiqi et al. 2010; van Dijk et al. 1993; Ye et al. 2004). For these reasons, intestinal levels of HETEs and HODEs were analyzed using LC-ESI-MS/MS. UFP exposure significantly increased 15-HETE, 12-HETE, 5-HETE, 13-HODE, and 9-HODE by 59.2 ± 5.7%, 22.8 ± 4.8%, 32.3 ± 15.5%, 44.2 ± 17.5%, and 78.5 ± 12.6%, respectively (*p* < 0.001 to *p* < 0.01, *n* = 4) (Figure 2), whereas administration of D-4F significantly reduced UFP-mediated increases in HETEs and HODEs (*p* < 0.01 to *p* < 0.05, *n* = 4) (Figure 2). Similar results were obtained in the second exposure study in which the mice were fed on normal chow diet (see Supplemental Material, Table S1). Thus, ambient UFP exposure is associated with an increase in oxidative metabolites of arachidonic and linoleic acids in *Ldlr*-null mice.

**Figure 3:**
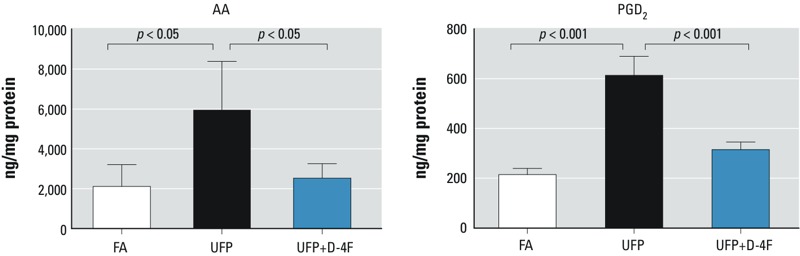
UFP exposure increased intestinal levels of AA and PGD_2_, which were attenuated by D-4F. Intestinal levels of AA and PGD_2_ were measured by LC-ESI-MS-MS in mice exposed to FA or UFP (exposure 1). UFP significantly increased intestinal levels of AA (*p* < 0.05, *n* = 4) and PGD_2_ (*p* < 0.001, *n* = 4), which were significantly attenuated by D-4F (*p* < 0.001, *n* = 4).

*UFP exposure increased intestinal levels of AA and PGD_2_*. Metabolites of AA further produce a number of proinflammatory lipid mediators such as prostaglandins and leukotrienes (Shimizu 2009). In a mouse model of diabetes, AA and PGD_2_ in the liver tissue were significantly higher than in the wild type (Morgantini et al. 2010). LC-ESI-MS/MS analysis demonstrated that UFP-exposed mice exhibited significant increases in AA and PGD_2_ by 180.0 ± 115.1% (*p* < 0.05, *n* = 4) and 185.7 ± 35.6% (*p* < 0.001, *n* = 4), respectively (Figure 3). Administration of D-4F significantly attenuated UFP-mediated effects (AA: *p* < 0.05, *n* = 4; PGD_2_: *p* < 0.001, *n* = 4). In corollary, UFP exposure to *Ldlr*-null mice fed on a normal chow diet resulted in similar changes to PGD_2_ but not AA (see Supplemental Material, Table S1).

**Figure 4:**
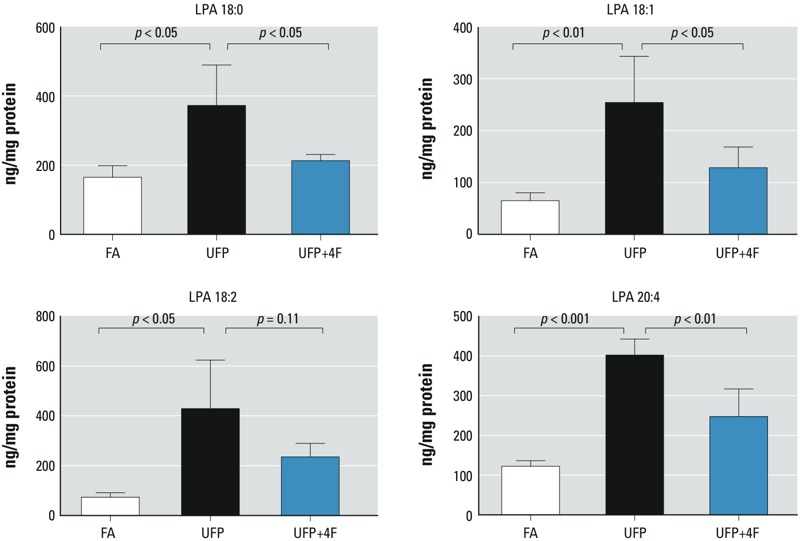
UFP exposure modulated intestinal levels of LPA. Intestinal levels of four different LPA were measured in mice exposed to FA or UFP (exposure 1). UFP exposure significantly increased all of the four measured LPA. Administration of D-4F significantly attenuated UFP-mediated increase of intestinal levels of LPA (18:0) (*p* < 0.05, *n* = 4), LPA (18:1) (*p* < 0.05, *n* = 4), and LPA (20:4) (*p* < 0.01, *n* = 4). D-4F also attenuated UFP-elevated intestinal levels of LPA (18:2) despite a *p*-value of 0.11.

*UFP exposure increased intestinal levels of LPA*. Phospholipids such as 1-palmitoyl-2-arachidonoyl-*sn*-glycerol-phosphatidylcholine (PAPC) release AA and LPA via phospholipase A_2_, and LPA is implicated in regulating the intestinal inflammatory responses (Sturm and Dignass 2002). Our LC-ESI-MS/MS analysis further revealed that UFP exposure significantly increased intestinal LPA [saturated LPA (length of carbon chain of fatty acid:number of double bonds in the carbon chain, 18:0): 125.6 ± 70.6%; unsaturated LPA (18:1): 291.1 ± 136.7%; LPA (18:2): 483.4 ± 266.2%; LPA (20:4): 229.2 ± 33.0%; *p* < 0.05 to *p* < 0.001, *n* = 4] (Figure 4). The administration of D-4F significantly mitigated the UFP-mediated increase in LPA (18:0) (*p* < 0.05, *n* = 4), LPA (18:1) (*p* < 0.05, *n* = 4), LPA (20:4) (*p* < 0.01, *n* = 4). Findings from the second exposure study reproduced similar results (see Supplemental Material, Table S1).

**Figure 5:**
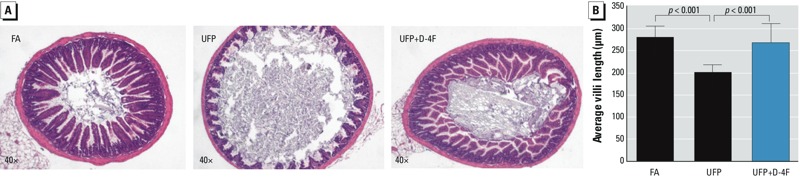
Villous morphological change in response to UFP exposure. (*A*) Cross-sections of mice ileum in response to FA, UFP, and UFP+D-4F were stained with H&E. (*B*) The villi of small intestine from UFP exposed mice were significantly shortened (*p* < 0.001, *n* = 4). D-4F administration significantly attenuated UFP reduction on villous lengths (*p* < 0.001, *n* = 4). Data are from exposure 1.

*Changes in the intestinal villus lengths in response to UFP exposure*. H&E staining indicated that villi of the ileum from UFP-exposed mice were significantly shortened as compared to control FA-exposed mice (FA = 280 ± 25 μm, UFP = 202 ± 17 μM, *p* < 0.001, *n* = 4) (Figure 5A,B). However, administration of D-4F significantly abrogated UFP-mediated reduction in villus lengths (Figure 5B). The second exposure study also recapitulated the changes in the villus morphology (data not shown), supporting the notion that UFP exposure induced epithelial damage to the intestinal villi.

**Figure 6:**
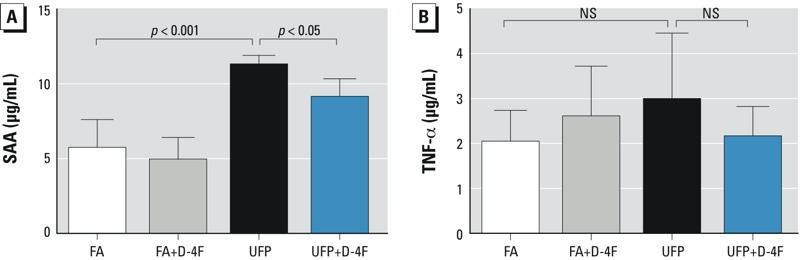
UFP exposure modulated the plasma levels of inflammatory markers. The plasma levels of SAA and TNF-α in mice on chow diet were measured (exposure 2). NS, no significant difference. (*A*) UFP increased SAA levels, which were significantly attenuated by administration of D-4F (*n *= 6). (*B*) TNF-α levels were not significantly elevated in mice on the normal chow diet (*n *= 6).

*Plasma measures of inflammation in response to UFP exposure*. UFP exposure promoted systemic inflammation as indicated by increased plasma levels of SAA in mice on both high-fat diet [first exposure study, as previously reported (Li et al. 2013), and chow diet (second exposure study) Figure 6A], and administration of D-4F significantly attenuated these effects (Li et al. 2013) (Figure 6A). Although UFP significantly increased plasma TNF-α levels in the fat-fed mice (Li et al. 2013), a similar trend in the chow-fed mice did not reach statistical significance (Figure 6B). Administration of D-4F had no significant effects on plasma TNF-α levels in both high-fat (Li et al. 2013) and chow-fed mice (Figure 6B).

**Figure 7:**
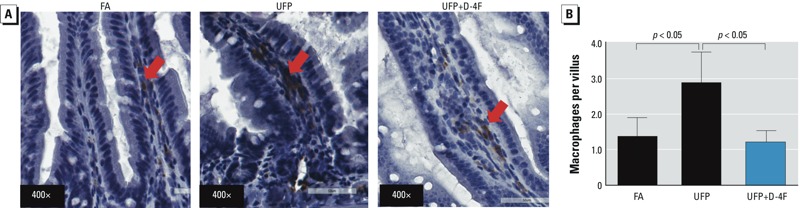
UFP exposure promoted intestinal inflammation. (*A*) Cross-sections of mice ileum were stained with antibody against F4/80 for macrophages (representative pictures, *n *= 4; arrows point to macrophages). (*B*) UFP exposure significantly increased the number of macrophages entered into the small intestine. This effect was significantly reversed by D-4F administration (*n *= 4). Data are from exposure 1.

Next, we performed immunohistochemistry staining for macrophages. Prominent F4/80-positive macrophage infiltration was observed in the lamina propria of intestinal villi. The average macrophages per intestine villus were 1.4 ± 0.52 for FA-exposed mice and 2.90 ± 0.86 for UFP exposed mice (*n* = 4, *p* < 0.05) (Figure 7). Administration of D-4F significantly abrogated UFP-mediated macrophage infiltrates (Figure 7B). Macrophage infiltration as quantified by the staining intensity was significantly increased in response to UFP exposure, and administration of D-4F significantly attenuated this effect (*p* < 0.01, *n* = 6) (see Supplemental Material, Figure S2A). Neutrophil infiltration was also significantly increased in response to UFP exposure (*p* < 0.01, *n* = 6) (see Supplemental Material, Figure S2B). Administration of D-4F revealed a trend toward a reduction in UFP-mediated intestinal neutrophil infiltration (*p* = 0.15, *n* = 6). Thus, UFP exposure altered the intestinal villus morphology (Figure 5), accompanied by an increase in the inflammatory responses, which were partially attenuated by D-4F.

On the contrary, there was no evidence for UFP-mediated induction of obvious pulmonary inflammation as determined by the lack of significant effects on the total cell count, cell differential, total protein, and lactate dehydrogenase and TNF-α levels in the bronchoalveolar lavage fluid (see Supplemental Material, Figure S3).

## Discussion

A number of emerging studies suggest that air pollutants may have an impact on the gastrointestinal tract. In the current study, ambient UFP promoted intestinal pro-oxidative and pro-inflammatory effects. The protective effect of D-4F, an antioxidant and anti-inflammatory peptide, supports the notion that lipid metabolism regulates UFP-mediated inflammatory responses (Navab et al. 2012). We provide new evidence that UFP exposure increased potentially oxidative lipid metabolites, namely AA, HETEs, HODEs, PGD_2_, and LPA, in the intestines. This evidence suggests new insights into the mechanisms underlying air pollution and gastrointestinal inflammatory responses.

Although elevated serum levels of HETEs and HODEs are implicated in atherosclerotic lesions (Funk and Cyrus 2001; Imaizumi et al. 2010), PM has been proposed to modulate lipid metabolism, including the production of HETEs and HODEs in the enterocytes via local and systemic pathways (Navab et al. 2012). In our second exposure study (see Supplemental Material, Table S1), UFP exposure significantly increased lipid metabolites: namely, 9- and 13-HODE, 12- and 15-HETE, PGD_2_ in the intestines, liver, and plasma, and LPA in intestines. Furthermore, UFP exposure mediated increase in LPA (Figure 4; see also Supplemental Material, Table S1), which is consistent with the previously reported association with intestinal inflammatory responses (Sturm and Dignass 2002). In this context, exposure to smaller particles favors systemic oxidative stress and inflammatory responses (Araujo and Nel 2009; Brook et al. 2010) partly via the oxidative metabolism of arachidonic and linoleic acid in the liver and intestines (Yin et al. 2013). Epidemiological and animal studies suggest the role of short-term ambient pollutant exposure was associated with nonspecific abdominal pain (Beamish et al. 2011). Although genome-wide association studies (GWAS) have identified over 160 genetic risk loci that predispose people to inflammatory bowel disease (IBD), these inherited factors were insufficient to account for the etiology of these chronic diseases (Aujnarain et al. 2013). Salim et al. (2014) have reported that urban ambient particulate matter ingested via contaminated food and water sources can alter gut microbiome and immune function, providing evidence on changes seen in intestinal barrier. Aujnarain et al. (2013) further addressed the increase in pediatric IBD, addressing environmental risk factors as the possible role in altering the intestinal microbiome. Although the focus of our studies was not on IBD, we provided new insights into the link between ambient UFP exposure and intestinal lipid peroxidation.

In response to tissue injury and stress, elevation of lipid metabolites, including those of arachidonic and linoleic acids, modulates inflammatory responses (Shimizu 2009). Beck-Speier et al. (2012) demonstrated that exposure to UFP increased 15-HETE in the alveolar macrophages using an allergic mice model. We recently observed that UFP exposure to *Ldlr*-null mice promoted atherosclerosis in parallel with an increase in plasma HETEs and HODEs (Li et al. 2013). Here, we demonstrated that UFP exposure increased intestinal free oxidative fatty acids and LPA with an implication in gastrointestinal inflammatory responses. We observed an altered intestinal villous morphology and an increase in intestinal macrophage and neutrophil infiltration, implicating both acute and chronic immune responses. Furthermore, D-4F significantly attenuated UFP-induced macrophage infiltration, suggesting that elevated levels of oxidative lipid metabolites modulate air pollution–mediated intestinal inflammatory responses. This finding is consistent with the reported association between the deficiency in cytosolic phospolipase A_2,_ a lipid metabolizing enzyme, and the development of intestinal ulcers and perforations (Adler et al. 2009). However, whether villus blunting is the result of inflammatory responses or changes in the lipid profiles remains undefined.

D-4F is an ApoA-I mimetic peptide synthesized from D-amino acids. It possesses both anti-inflammatory and antioxidant properties to mitigate the extent of atherosclerosis via the HDL anti-inflammatory function (Morgantini et al. 2011; Navab et al. 2002). We previously demonstrated that UFP exposure modulated lipid metabolism and antioxidant capacity of HDL associated with an increase in atherosclerosis lesion size, which were significantly attenuated by D-4F administration (Li et al. 2013). In this study, UFP-mediated increases in intestinal HETEs, and HODEs as well as other lipid metabolites were also significantly reduced by D-4F. Importantly, reductions in oxidative lipid metabolites were associated with a decrease in morphological changes and inflammatory responses in the small intestine, suggesting that UFP indeed mediated intestinal injury through lipid peroxidation in these metabolites. Navab et al. (2012) reported that D-4F accumulates in the intestines regardless of the route of administration; and D-4F harbors a strong binding capacity to oxidized fatty acids such as HETEs and HODEs (Van Lenten et al. 2008). In our second exposure study, D-4F administration significantly reduced UFP-mediated increase in lipid metabolites (HODEs, HETEs) in the intestines. D-4F further reduced PGD_2_ and LPA in the intestines (see Supplemental Material, Table S1). Thus, D-4F administration is implicated in attenuating UFP-mediated increases in oxidative products of fatty acids in the intestines. This finding leads to a future investigation into a potential link between atherosclerosis and gastrointestinal disorders.

Additional hematological elements may contribute to an increase in free HETEs and HODEs. Erythrocytes may account for the source of the HETEs and HODEs (Navab et al. 2012). Although high-fat diet may also account for the source, the diet used to feed the *Ldlr*-null mice in our studies contained low levels of free HETEs and HODEs (Navab et al. 2012). For this reason, enzymatic or nonenzymatic oxidative metabolites of arachidonic and linoleic acids were mainly the sources of intestinal HETEs and HODEs (Natarajan and Nadler 2004). However, the precise mechanisms whereby exposure to air pollutants promotes intestinal oxidative products of fatty acids and the role of the gut in mediating metabolic syndrome and hepatic disease through lipid metabolites warrant further investigation.

An interesting observation in the study was the effect of diet in modulating serum TNF-α levels in *Ldlr*-null mice exposed to UFP. UFP exposure in mice under high-fat diet significantly increased TNF-α levels (Li et al. 2013), whereas the effect under normal chow diet on TNF-α levels was statistically insignificant, implicating synergistic effects between UFP and high-fat diet. In both cases, administration of D-4F did not significantly reduce TNF-α levels, further suggesting that UFP may up-regulate TNF-α expression via an oxidized lipid-independent mechanism.

Increasing evidence supports the potential mechanisms underlying UFP-induced oxidative and inflammatory effects in the intestines. Particles are effectively cleared from the lungs and transported to the intestinal tract by mucociliary clearance (Kreyling et al. 1999; Möller et al. 2004). During this process, a large fraction of inhaled pollutants will be ingested and rapidly enter into the intestines. Particles can also be ingested via food and water sources (Beamish et al. 2011) and, in the case of our experiments, by grooming of the animal fur coat where particles could have been deposited after inhalation. Interestingly, we did not observe evidence of obvious inflammatory effects in the lungs in our second experiment (see Supplemental Material, Figure S3), which suggests that a good number of UFP could have primarily accessed the gastrointestinal tract, by-passing the lungs, or that the intestinal mucosa could be more sensitive to the UFP proinflammatory effects than cells in the lungs. This is consistent with our previous studies, where we have not observed obvious pulmonary inflammatory responses in *ApoE*-null mice exposed to ultrafine concentrated ambient particles for 5 weeks (Araujo et al. 2008) or diesel exhaust for 2 weeks (Yin et al. 2013). It is also consistent with the general recognition that fine particles are cleared from the respiratory tract via mucociliary clearance to make their way to the gut (Beamish et al. 2011; Salim et al. 2014). However, we cannot rule out the activation of immune cells in the lungs such as alveolar macrophages, or molecular pathways that could be implicated in the induction of systemic effects. The precise mechanisms remain to be defined.

One of the limitations of the present study was the end point data. A time course study would provide insights into the interactions between different organ systems—pulmonary, digestive and/or cardiovascular systems. We analyzed a number of lipid metabolites in response to UFP exposure and D-4F administration. Power analyses revealed that the effect sizes ranged from 1.28 to 5.15 and power values from 14% to 99%. Although some of our observations marginally missed statistical significance, data associated with a power value < 80% suggest the need to increase the sample size beyond our *n* = 6 per group. Another limitation of the study was the use of *Ldlr*-null mice to elucidate the effects of UFP in the digestive system. Similar to *ApoE*-null mice, *Ldlr*-null mice are used as model of atherosclerosis to investigate the mechanisms underlying risk factors and the initiation of atherosclerosis, but the *Ldlr*-null strain is dependent on diet-induced hyperlipidemia (Tangirala et al. 1995). Nevertheless, the use of the *Ldlr-*null strain provided the first evidence linking UFP-induced atherosclerosis with intestinal lipid peroxidation and subsequent inflammatory responses. Thus, these novel findings from *Ldlr*-null mice provide the framework to study the effects of UFP exposure in a gastrointestinal model.

## Conclusions

*Ldlr*-null mice exposed to UFP developed an increase in the intestinal levels of oxidized fatty acids and LPA, accompanied with both altered villus morphology and inflammatory cell infiltration. D-4F administration mitigated these effects. These findings provide the potential mechanisms underlying metabolites of lipid peroxidation and gastroenterological inflammatory responses, underscoring the role of D-4F as an antioxidant peptide to mitigate air pollutant-mediated atherosclerotic lesions and gastrointestinal injury.

## Supplemental Material

(1.3 MB) PDF
