# Supplementary material for: Effect of Exposure to Atmospheric Ultrafine Particles on Production of Free Fatty Acids and Lipid Metabolites in the Mouse Small Intestine
Source: Environ Health Perspect. 2014 Aug 29;123(1):34–41. doi: 10.1289/ehp.1307036 (PMC4286268; doi:10.1289/ehp.1307036)
Supplement: (1.3 MB) PDF [file ehp.1307036.s001.508.pdf]

Environmental Health Perspectives doi:10.1289/ehp.1307036

Supplemental Material

Effect of Exposure to Atmospheric Ultrafine Particles on Production of Free Fatty Acids and Lipid Metabolites in the Mouse Small Intestine

Rongsong Li, Kaveh Navab, Greg Hough, Nancy Daher, Min Zhang, David Mittelstein, Katherine Lee, Payam Pakbin, Arian Saffari, May Bhetraratana, Dawoud Sulaiman, Tyler Beebe, Lan Wu, Nelson Jen, Eytan Wine, Chi-Hong Tseng, Jesus A. Araujo, Alan Fogelman, Constantinos Sioutas, Mohamed Navab, and Tzung K. Hsiai

Materials and Methods

BALF collections and cell analyses

The collection and analysis of bronchoalveolar lavage fluid (BALF) were done according to Zhang et al (Zhang et al. 2008) with minor modifications. Briefly, mice were euthanized and their tracheas were exposed. An 18 gauge needle (1 inch length) attached to a 3 mL syringe was inserted into the trachea to flush the lungs multiple times using 1.5 mL of cold phosphate buffered saline (PBS) with 0.5 mM ethylenediaminetetraacetic acid (EDTA), which was immediately placed on ice after collection. The lavage fluid from each mouse was collected in an Eppendorf tube and used for cell counts, cell differentials, analysis of proteins including total protein concentration, LDH release and TNF-α levels in BALF. Cell counts were performed using a hemocytometer. Cytospin slides were made using 100 μL of the BALF, which were added to cytospin funnels and centrifuged at 600 rpm for 5 minutes to form the cell layer on the cytospin slides. The slides were stained using a Hema 3 staining kit (Fisher Scientific, catalog no. 22-122-911), with minor modifications to the manufacturer's instructions. Briefly, the slides were placed in the fixative for 2 minutes, stained in Solution I for 5 minutes, dipped five times in Solution II, and then dipped multiple times in cold water to rinse away the excess stain. Cell differentials were performed under brightfield microscropy- 200 cells were counted per slide and noted as a macrophage, a neutrophil, or a lymphocyte.

BALF chemical and cytokine analyses

BALF total protein concentration was measured using a Bradford protein assay kit (Biorad) according to the manufacturer's instructions. The assay was done in duplicate. LDH content was measured by CytoTox-ONE homogeneous membrane integrity assay (Promega) according to the manufacturer's instructions. 50 µl of BALF was used for each assay. The assay was done in duplicate. TNF-α levels were measured by mouse TNF-α ELISA MAX Deluxe sets (Biolegend) according to the manufacturer's instructions. 100 µl of BALF from each mouse was used for each assay. The assay was done in triplicate.

References

Zhang, X., Goncalves, R. and Mosser, D. M. The isolation and characterization of murine macrophages. Curr Protoc Immunol 2008 Chapter 14: Unit 14 1. PMC2834554.

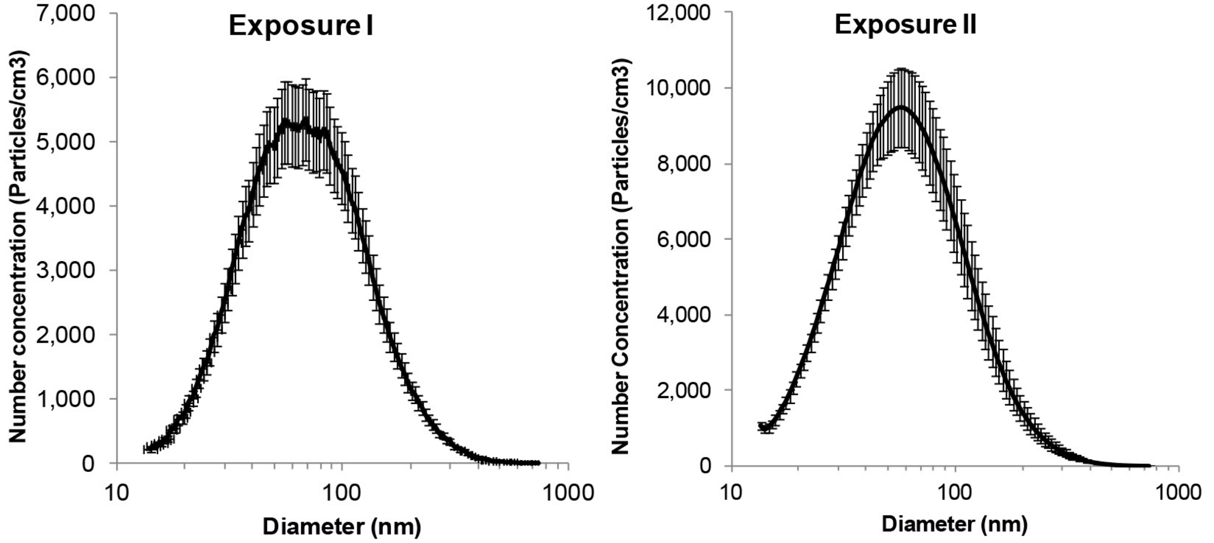

Exposure I	Average±standard error		Exposure II	Average±standard error
Median diameter	66.6±4.7		Median diameter	57.0±0.66
Mean diameter	81.6±5.8		Mean diameter	70.6±0.75
Mode diameter	68.5±4.8		Mode diameter	56.6±0.82
Total conc. (#/cm^3)	2.4E^{04}±1.7E^{04}		Total conc. (#/cm^3)	4.5E^{04}±3.1E^{04}

Figure S1. Size distribution and number concentrations of UFP. Between the exposures I and II, the mode (68.5 vs 56.6 nm), median (66.6 vs 57.0 nm) and mean diameters (81.6 vs 70.6 nm)of UFP were similar.

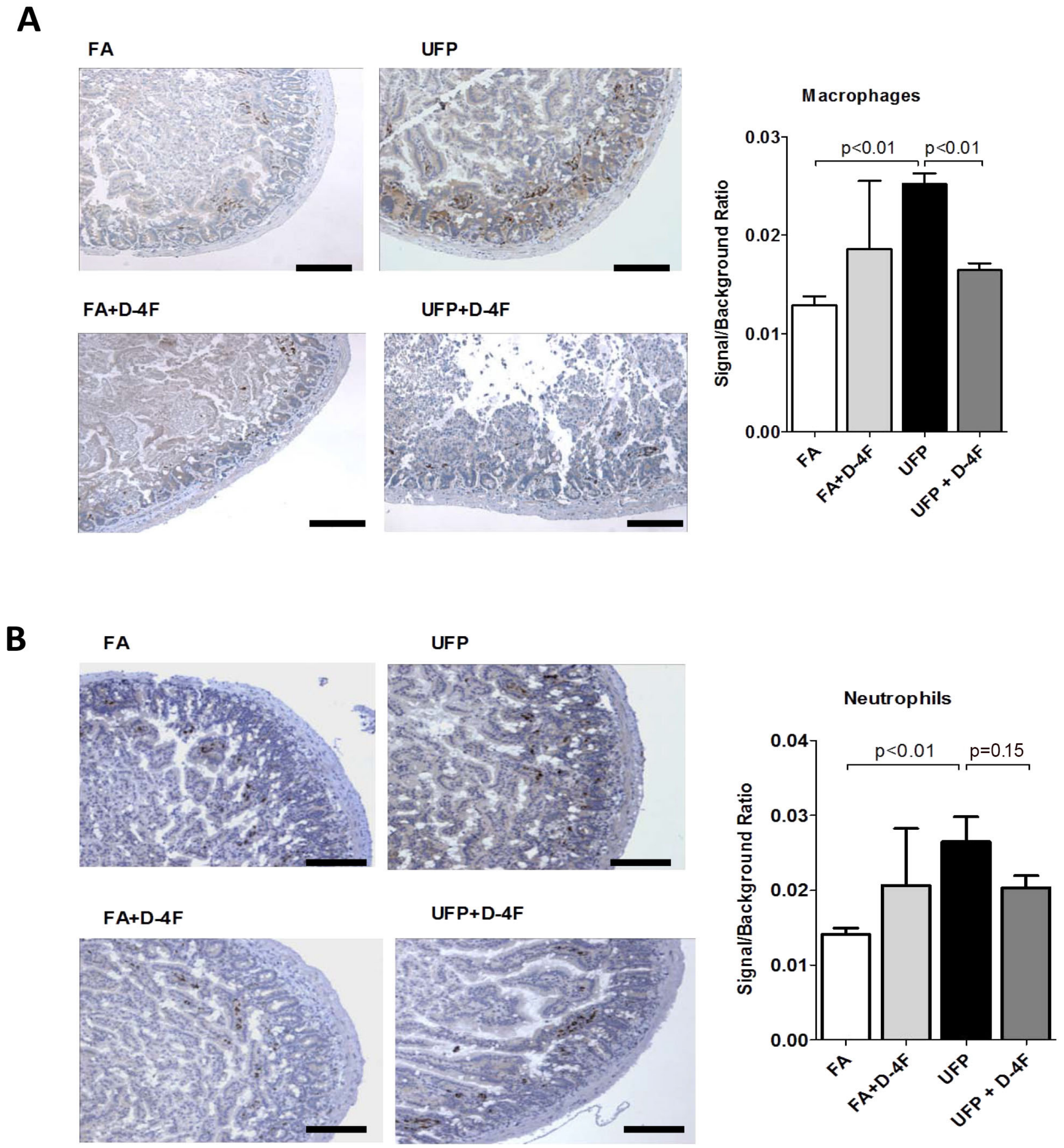

Figure S2. UFP exposure promoted intestinal inflammation. Cross sections of mice ileum from exposure II were stained with (A) antibody against F4/80 for macrophages and (B) antibody against Ly6G for neutrophils. The left panels are representative photographs. The right bar graphs revealed the staining intensity as quantified by using the NIH ImageJ software. UFP exposure significantly increased the numbers of macrophage and neutrophil infiltration ($p < 0.01$, n=6). D-4F administration significantly attenuated UFP-stimulated macrophage infiltration ($p < 0.01$, n=6). D-4F did not significantly reduce the neutrophil infiltration ($p = 0.15$, n=6). Scale bar = 200 μm.

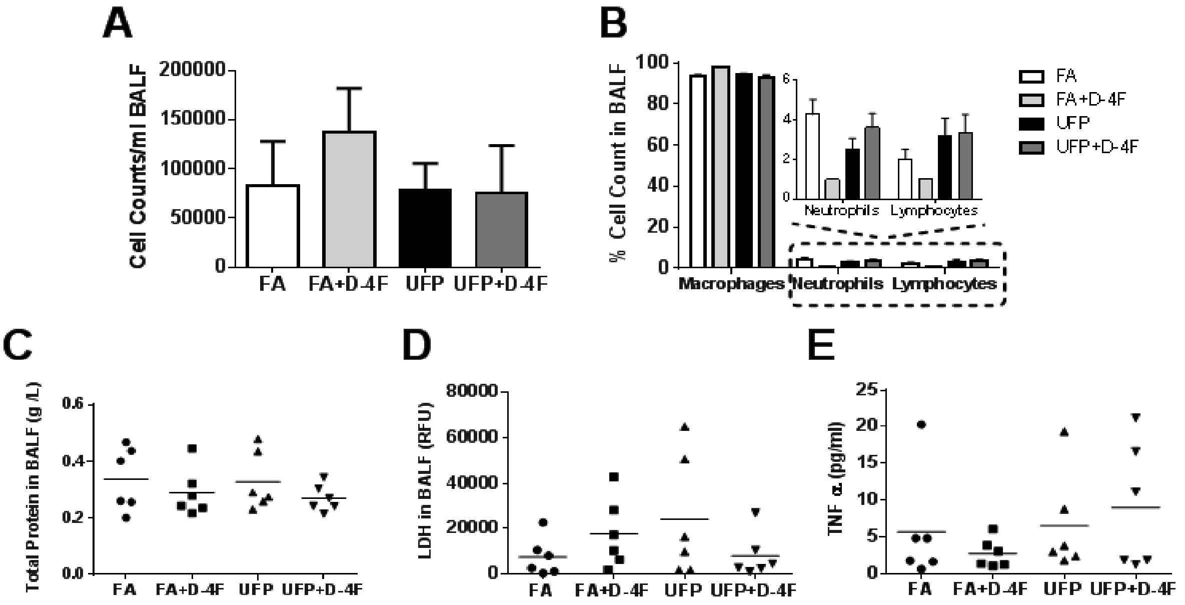

Figure S3. Characterization of BALF collected in exposure II. There were no significant differences in (A) Total cell counts, (B) Cell differentials, (C) Total protein, (D) LDH and (E) TNF-α in the BALF. Assessment was performed in samples from individual mice, n=6/group for all metrics except for the cell differentials (B) since some cytospin slides were not quantifiable (n=1,2 for FA+D4F and FA groups, respectively and n=5,6 for the UFP and UFP+D4F groups, respectively). Statistical analyses were performed by one-way ANOVA.

Table S1. Concentration of lipid metabolites in tissues of exposure II.

Tissue	9-HODE	13-HODE	5-HETE	12-HETE	15-HETE	AA	PGD2	LPA 18:0	LPA 18:1	LPA 18:2	LPA 20:4
**Intestine (ng/mg protein)**											
FA	365 ± 22	171 ± 27	44.14 ± 7.50	1137 ± 182	127 ± 20	4553 ± 527	107 ± 8.4	113 ± 13	45.09 ± 5.15	63.89 ± 7.21	29.85 ± 3.15
FA+D-4F	314 ± 50	137 ± 15	29.97 ± 4.79	877 ± 140	98 ± 16	3429 ± 422	89 ± 4.9	85 ± 11	34.75 ± 3.48	50.60 ± 5.06	23.73 ± 2.37
UFP	464 ± 66*	216 ± 37#	53.88 ± 7.70#	1469 ± 175*	163 ± 22*	5582 ± 493	131 ± 9.8*	139 ± 12*	58.39 ± 5.16*	84.89 ± 7.43*	39.63 ± 3.22*
UFP+D-4F	347 ± 36**	169 ± 25**	41.22 ± 6.25**	1161 ± 157**	129 ± 25**	4935 ± 436	116 ± 9.2**	123 ± 11	49.17 ± 4.34**	71.28 ± 6.16**	33.41 ± 2.88**
**Liver (ng/mg protein)**											
FA	190 ± 17	251 ± 31	17.05 ± 1.40	157 ± 31	12.20 ± 3.12	9031 ± 728	20.53 ± 3.24	NM	NM	NM	NM
FA+D-4F	183 ± 14	242 ± 26	16.47 ± 0.94	151 ± 27	11.77 ± 2.81	8727 ± 549	19.83 ± 2.85	NM	NM	NM	NM
UFP	254 ± 31*	334 ± 44*	25.15 ± 5.44*	237 ± 39*	19.51 ± 4.18*	11729 ± 94*	28.33 ± 4.6*	NM	NM	NM	NM
UFP+D-4F	219 ± 22	282 ± 30	19.98 ± 1.54**	180 ± 40**	14.42 ± 3.40**	10662 ± 859	25.75 ± 4.20	NM	NM	NM	NM
**Plasma (ng/mL)**											
FA	7.26 ± 1.17	8.24 ± 1.32	1.83 ± 0.29	188 ± 30	2.86 ± 0.46	4322 ± 695	1.88 ± 0.30	NM	NM	NM	NM
FA+D-4F	7.24 ± 1.32	8.10 ± 0.84	1.89 ± 0.52	184 ± 18	2.76 ± 0.58	4192 ± 445	2.06 ± 0.91	NM	NM	NM	NM
UFP	9.04 ± 1.38	10.23 ± 1.51	2.40 ± 0.55	246 ± 35*	3.66 ± 0.62*	5278 ± 837	2.46 ± 0.56*	NM	NM	NM	NM
UFP+D-4F	7.26 ± 1.17**	8.24 ± 1.32**	1.83 ± 0.29**	188 ± 30**	2.86 ± 0.46**	4322 ± 695**	1.88 ± 0.30**	NM	NM	NM	NM

*UFP vs. FA, $p < 0.05$, n=6; **UFP+D-4F vs. UFP, $p < 0.05$, n=6. NM = not measured.
